# Global termite methane emissions have been affected by climate and land-use changes

**DOI:** 10.1038/s41598-023-44529-1

**Published:** 2023-10-11

**Authors:** Akihiko Ito

**Affiliations:** 1 The University of Tokyo, Tokyo, Japan; 2 National Institute for Environmental Studies, Tsukuba, Japan; 3 Japan Agency for Marine-Earth Science and Technology, Yokohama, Japan

**Keywords:** Carbon cycle, Ecosystem ecology, Climate-change mitigation

## Abstract

Termites with symbiotic methanogens are a known source of atmospheric methane (CH_4_), but large uncertainties remain regarding the flux magnitude. This study estimated global termite CH_4_ emissions using a framework similar to previous studies but with contemporary datasets and a biogeochemical model. The global termite emission in 2020 was estimated as 14.8 ± 6.7 Tg CH_4_ year^−1^, mainly from tropical and subtropical ecosystems, indicating a major natural source from upland regions. Uncertainties associated with estimation methods were assessed. The emission during the historical period 1901–2021 was estimated to have increased gradually (+ 0.7 Tg CH_4_ year^−1^) as a result of combined influences of elevated CO_2_ (via vegetation productivity), climatic warming, and land-use change. Future projections using climate and land-use scenarios (shared socioeconomic pathways [ssp] 126 and 585) also showed increasing trends (+ 0.5 to 5.9 Tg CH_4_ year^−1^ by 2100). These results suggest the importance of termite emissions in the global CH_4_ budget and, thus, in climatic prediction and mitigation.

## Introduction

Methane (CH_4_) is a potent greenhouse gas in the global climate system and a short-lived climate forcer of local air quality^1,2^. CH_4_ accounts for about 20% of the historical air temperature rise, and during the next decades, its contribution to global warming is predicted to be comparable to that of carbon dioxide^3^. Regulating the atmospheric CH_4_ concentration is therefore pivotal for climate management, including the accomplishment of the long-term goals of the Paris Agreement and the Global Methane Pledge^4^.

Elucidating global and regional greenhouse gas budgets is essential in both scientific and social contexts for making predictions and deploying effective mitigation options. There remain, however, considerable uncertainties in the current understanding of the CH_4_ budget because of the various and heterogeneous natural sources and complicated human interventions^5,6^. For example, because there is still no consensus about the mechanisms of the decadal changes in the atmospheric CH_4_ concentration^7^, it is difficult to separate natural and human influences. Many studies have focused on detecting anthropogenic CH_4_ emissions, including point sources and intermittent leakages^8^, and the recent progress in satellite observations and surface flux inversion techniques is encouraging. Among natural sources, wetlands have been focused of biogeochemical and climatic studies, which have made considerable progress in field flux measurement and model development^9,10^. However, evaluating other natural emissions such as wildfires, ruminants, and termites distributed heterogeneously over Earth's surface is still difficult.

Termites (Isoptera) are soil, wood or fungi feeding insects, who are known for their advanced social structure (e.g., workers, soldiers, and queens). Most termites have symbiotic microbes (Methanogens archaea) in their guts that enable them to digest lignocellulose, which are a known source of CH_4_^11,12^. Because of this biogeochemical characteristics, termites have attracted the attention from scientists for many years. Gas exchange with the atmosphere, measured by using chamber and collar techniques, has been found to occur mainly at ‘hot spots’ around nests and mounds. Because of the intensity of this source, several studies have attempted to evaluate termite CH_4_ emissions in a global biogeochemical context (Table S1). For example, Zimmerman et al.^13^ estimated termite emissions by using termite densities and biomass consumption rates, measured in a limited number of laboratory experiments, whereas Fung et al.^14^ developed 1° × 1° global maps of surface CH_4_ sources including termites and sinks to simulate the global CH_4_ cycle with an atmospheric model. In addition to biomass, Sugimoto et al.^15^ used stable carbon isotopic signatures to estimate CH_4_ production and oxidation rates at termite mounds. The results of these early studies suggest that termite emissions constitute a substantial component of the global CH_4_ cycle, and that biogeographically, they occur mainly in low-latitude woodlands and forests^16,17^. These studies used termite biomass density and emissions factors (gas emission rate per unit biomass and time) to estimate termite CH_4_ emissions, but the high heterogeneity of the emissions makes it difficult to conduct spatially representative measurements. Therefore, there remain large uncertainties in present estimations of global termite CH_4_ emissions (Supplementary Table S1). Moreover, little is known about temporal changes in termite emissions, which may be affected by global and local environmental changes. Revisiting global termite CH_4_ emissions and assessing their temporal variation would improve our understanding of the global CH_4_ cycle and, eventually, help in making climatic projections that include biogeochemical feedback.

The objectives of this study were thus to (1) revisit the estimation of global termite CH_4_ emissions by using modern data and methods, and (2) investigate temporal changes and their driving factors by conducting simulations for historical and future periods. Additionally, this study explored the range of the estimation uncertainty by analyzing estimates derived by using different emission factors and methods.

## Results and discussion

### Global total emissions

**Figure 1:**
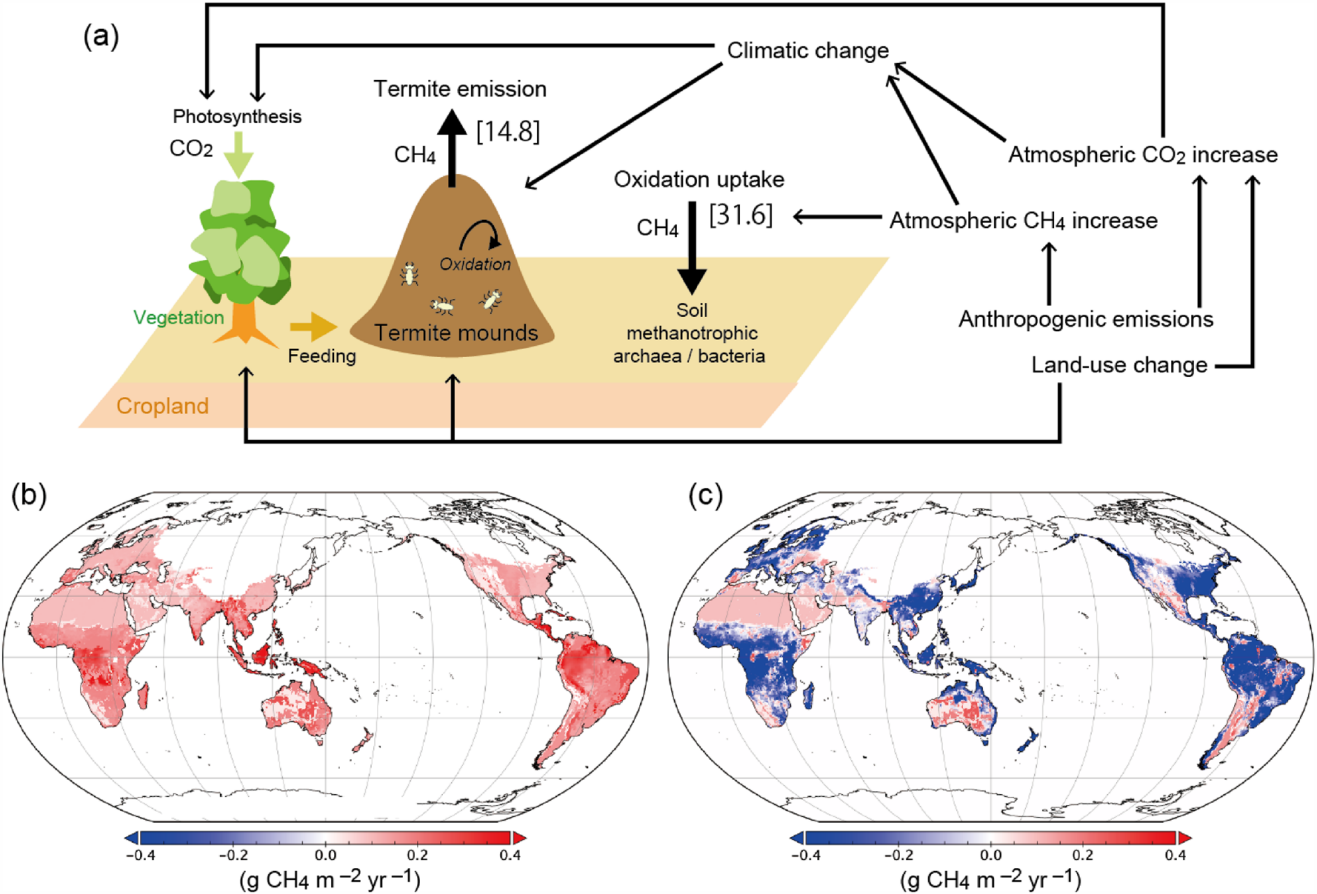
Upland CH_4_ budget including termite emissions. (**a**) Schematic diagram of the upland CH_4_ budget, including termite emissions. Numbers in square brackets indicate the global total CH_4_ flux estimated by this study for the year 2020 (Tg CH_4_ yrar^–1^). Distributions of (**b**) termite CH_4_ emissions and (**c**) the net CH_4_ flux, including soil oxidation uptake estimated by a process-based model (maps generated by Panoply 5.2.9, https://www.giss.nasa.gov/tools/panoply/).

Termite CH_4_ emissions were estimated globally using a framework similar to previous studies (i.e., emission = termite density × emission factor; see “Methods and data”) and contemporary datasets. This study took account of influential factors such as climate, land-use, and vegetation photosynthetic productivity (Fig. 1a) during the historical (1901–2020) and future (2021–2100) periods. Climate and land-use were derived from existing datasets (Supplementary Fig. S1), and vegetation productivity was obtained from a simulation of terrestrial carbon cycle with a process-based biogeochemical model (VISIT).

The land area of potential termite habitat was estimated using an empirical temperature threshold (see “Methods and data”) to be 92.9 × 10^6^ km^2^ in 2020, mostly in Africa, Australia, and South America, as well as large parts of Asia, Europe, and North America. Vast regions without termite habitat were seen in northern Eurasia and North America. The actual habitat area, including a restriction to take account of land use for agriculture, was estimated as 79.8 × 10^6^ km^2^. By estimating empirically using model-simulated GPP in tropical ecosystems and assuming termite densities per habitat area in non-tropical ecosystems (see “Methods and data”), total termite biomass was estimated. This estimate, 122.3 Tg dry weight, is comparable to that of Rosenberg et al.^18^, who used thousands of measurement data to derive a total of 300 Tg (uncertainty range, 100–500 Tg) for underground (soil) and aboveground arthropods, of which 40%, or 120 (40–200) Tg, are Isoptera (termites). The results of Rosenberg et al. and this study imply that total termite biomass is larger than that of Formicidae (ants) and comparable to that of humans (100 Tg dry weight)^19^.

By using emission factors derived from a recently published dataset by Zhou et al.^20^ and termite biomass density mentioned above, the global total termite CH_4_ emission was estimated as 14.8 ± 6.7 Tg CH_4_ year^−1^ (mean ± standard deviation of random-sampling ensembles; see Statistical analysis of uncertainty in “Methods and data” section). This value is intermediate among those reported by previous studies (Table S1) and close to a relatively new independent estimate by van Asperen et al.^21^ of 14. 96 Tg CH_4_ year^−1^, based on observed tropical emissions data from Amazonia. It is slightly higher than the estimate adopted in the synthesis of the global CH_4_ budget of the Global Carbon Project: 9 [3–15] Tg CH_4_ year^−1^ (Ref.^5^). These differences among studies are discussed later. At this point, it is sufficient to note that we need to be careful about how emissions and related parameters are defined. As reported by Nauer et al.^22^, about half (20–80%) of the CH_4_ produced by termites may be oxidized within the mounds without reaching the atmosphere; therefore, the use of emission factors obtained from isolated termites (e.g., in a cuvette) is likely to cause overestimation of emissions to the atmosphere. Note that the present study did not select data by observation method. Nonetheless, clearly, the role of termite emissions in the CH_4_ budget is remarkable, especially those from upland regions. Because of methanotrophic oxidation, aerobic soils are a sink of atmospheric CH_4_ and estimated with a process-based model (VISIT, see “Methods and data”) to be 31.6 Tg CH_4_ year^−1^ from the termite-inhabited area in 2020 (Fig. 1b). Termite emissions likely offset about 47% of the absorption flux at landscape or larger scales. Note that the amount of offset varies among locations and through time, as described later.

### Spatial distributions

Spatial distributions of termite density estimated in this study (Supplementary Fig. S2) appear to be qualitatively comparable to those of termite diversity^16,17^. Termite CH_4_ emission intensity varied spatially from < 0.05 g CH_4_ m^−2^ year^−1^ in deserts to > 0.2 g CH_4_ m^−2^ year^−1^ in tropical forests in Africa, Southeast Asia, and South America (Fig. 1b), where high termite density was assumed (Fig. S2). The global pattern estimated in this study is roughly comparable to that obtained by Fung et al.^14^ (Supplementary Fig. S4). Regionally, Africa and South America accounted for about 55% of the total emission (4.5 and 3.5 Tg CH_4_ year^−1^, respectively) (Supplementary Fig. S3). Observations by field studies have shown similarly high termite CH_4_ emissions from tropical biomes. For example, Martius et al.^23^ conducted observations at wood-feeding termite nests in Amazonia and obtained comparable fluxes (~ 0.18 g CH_4_ m^−2^ year^−1^)^24^. Brümmer et al.^25^ reported termite emission in the savanna of West Africa to be about 0.25 g CH_4_ m^−2^ year^−1^; in their study, croplands were a net CH_4_ sink because soil uptake was larger than termite emission there.

Upland ecosystems such as grasslands and deserts can absorb atmospheric CH_4_ due to soil oxidation by methanotrophs. This study indicated that termite emissions are a substantial source and influence the upland CH_4_ budget, although other sources such as biomass burning are in a comparable magnitude and sometimes influential^26^. As a result of the heterogeneous distribution of these fluxes, it was shown that upland ecosystems can be both a net sink and a net source, depending on relative intensities of soil oxidation and termite emission (Fig. 1c). Such spatial heterogeneity is, although its area-based intensity is weaker than wetlands, important when interpreting and evaluating the upland CH_4_ budget especially using atmospheric observation data.

### Historical variability

**Figure 2:**
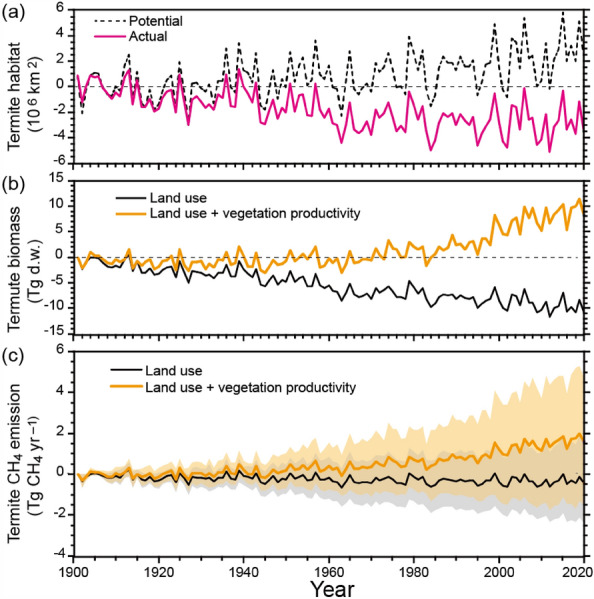
Temporal change in the estimated global termite CH_4_ emissions. (**a**) Potential and actual termite habitat areas, (**b**) total termite biomass in dry weight, and (**c**) total termite CH_4_ emissions. The shading shows standard deviation ranges obtained from 1000 ensemble calculations using randomly sampled emission factors.

During the historical period, global termite emissions were estimated to have gradually increased from 13.1 ± 0.1 Tg CH_4_ year^−1^ in the 1900s (1901–1910) to 14.8 ± 0.2 Tg CH_4_ year^−1^ in the 2010s (2011–2020) (mean ± standard deviation of interannual variability). The increase was associated with land-use, climate, and atmospheric changes, and could be attributable to changes in the habitat area and termite biomass. Potential (temperature-limited, black dotted line in Fig. 2a) termite habitat expanded in temperate to boreal regions as a result of climatic warming (~ 2.9 × 10^6^ km^2^). In contrast, actual habitat (also impacted by land use, red line in Fig. 2a) was estimated to decrease by 2.8 × 10^6^ km^2^, mainly because of conversion from natural vegetation to croplands. Termite biomass was estimated to decrease when considering only land use and habitat loss (black line in Fig. 2b). When including the effects of atmospheric CO_2_ increase and resultant fertilization on vegetation productivity (on average by 29%, estimated by the VISIT model), termite biomass was estimated to increase by 31 Tg dry weight (yellow line in Fig. 2b). Eventually, these factors explain the increase of termite CH_4_ emissions as shown by a difference between black and yellow lines of Fig. 2c.

The overwhelming increase in anthropogenic CH_4_ emissions during the historical period (> 100 Tg CH_4_ year^−1^)^27^ can make it difficult to detect the impact of the change of termite emissions on the atmospheric CH_4_ concentration. Nevertheless, the increased termite emissions may substantially influence the CH_4_ budget of upland areas, which cover a vast land area. Note, however, that soil CH_4_ oxidation was estimated to increase even more rapidly than termite emissions because of the elevated atmospheric CH_4_ concentration (nearly doubling from the 1910s to 2010s, estimated by the VISIT model).

### Projected emissions

**Figure 3:**
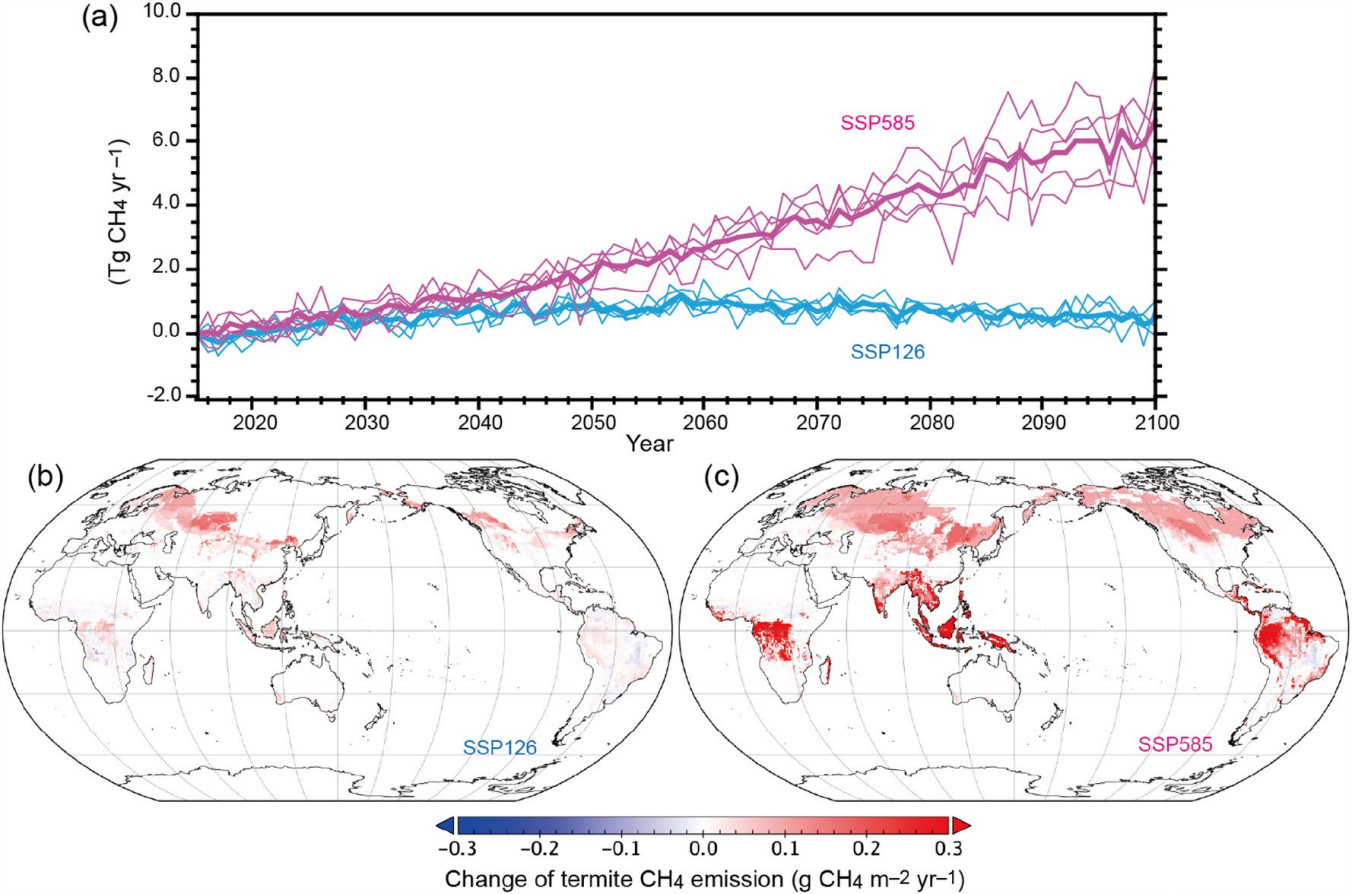
Projected global termite CH_4_ emissions. (**a**) Interannual variability under the ssp126 and ssp585 scenarios using five climate projections. Thin lines show individual climate model results, and thick lines show their mean. Distribution of the estimated changes for (**b**) ssp126 and (**c**) ssp585 from the 2010s to the 2090s (maps generated by Panoply 5.2.9, https://www.giss.nasa.gov/tools/panoply/).

The projections of termite CH_4_ emissions indicated that they will increase, with the pattern of increase dependent on the future scenario (Fig. 3). By the 2090s, under a mitigation-oriented scenario (ssp126), termite emissions were estimated to increase by 0.5 Tg CH_4_ year^−1^ (0.2–0.7 Tg CH_4_ year^−1^, depending on the climate scenarios), whereas under an adaptation-oriented scenario (ssp585), the estimated increase was 5.9 (4.8–7.0) Tg CH_4_ year^−1^. In the ssp226-based estimation, termite emissions showed a broad peak around the 2050s and then decreased gradually. This overshoot pattern is apparently comparable to the pattern of the atmospheric CO_2_ concentration in the scenario, which leads to a corresponding variation in vegetation productivity^3^. In contrast, the estimated termite CH_4_ emissions under the ssp585 scenario showed steady increases, again in parallel with the atmospheric CO_2_ level and associated climatic change. The differences among the climate projections by the five climate models were small in both scenarios compared with the difference between the ssp126 and ssp585 scenarios.

In the ssp126-based estimation, termite CH_4_ emissions increased mainly in northern temperate to boreal regions, where termite habitat is currently limited by cold temperatures (Fig. 3b), and emissions in tropical and subtropical areas were relatively unaffected. In the ssp585-based estimation, termite CH_4_ emissions were estimated to increase not only in the temperate to boreal but also in tropical to subtropical regions. The increases in temperate to boreal regions of Northern Europe, Eurasia, and North America were associated with the northward expansion of termite habitat, whereas the increases in the tropical to subtropical regions of Africa, Southeast Asia, and South America were associated with increases in vegetation productivity. Globally, these increases completely offset the decreased emissions in subtropical areas caused by land-use conversion from natural vegetation to croplands (e.g., in savanna regions of Africa and South America).

The projected climate change will affect termite distribution and activities, leading to various (i.e., both positive and negative) indirect impacts on ecological processes such as carbon and nutrient cycling. To date, few studies have attempted to predict termite activities including CH_4_ emissions, although future emissions from other natural sources and their climatic feedback have been explored^28^. Zanne et al.^29^ estimated future changes in termite-induced woody decay by using climate scenarios similar to those used in the present study. Their conclusion that termite functions in the terrestrial carbon cycle will be enhanced in the future is consistent with the findings of the present study, although they put more focus on the sensitivity of emissions to the temperature change (> 6.8 times per 10 °C warming). If termite feeding activities are as sensitive to temperature as implied by Zanne et al.^29^, then the future increase of termite CH_4_ emissions estimated in this study, 40% in the ssp585-based estimation for the 2090s, could be much larger. By contrast, Buczkowski and Bertelsmeier^30^, using a species distribution model, suggested that habitat expansion of invasive termites may occur, for example, in Europe. Further studies should examine future termite CH_4_ emissions both by considering physiological mechanisms and by conducting continuous, extensive field observations.

### Estimation uncertainty

Previous estimates of the global termite CH_4_ emissions range from 1 to 152 Tg CH_4_ year^−1^ (summarized in Supplementary Table S1). This wide disparity, especially in the early studies, is apparently associated with biases stemming from the use of a limited number of observations that could not adequately represent the vast area of termite habitats. In addition, the mechanistic understanding of the factors that determine the spatial and temporal variation of the emissions was insufficient.

To address the estimation uncertainty, the results of several supplementary estimations were compared (see emission maps in Supplementary Fig. S5). The estimate described in the previous sections, using land use- and vegetation productivity-based termite density and emission factors from Zhou et al.^20^ (Fig. 1b and Fig. S5d), was referred to hereafter as the control. First, when emission factors specific to each land-cover type, derived from Sanderson were used instead of random sampling from the dataset, the total global emission in 2020 was estimated as 17.3 ± 2.6 Tg CH_4_ year^−1^, that is, 18% higher than the control estimate of this study. This higher estimate is attributable to the high emission factor (5.9 µg CH_4_ g^−1^ termite h^−1^) obtained from Sanderson and applied to all tropical forests (see Fig. S5 for the spatial pattern). Second, when termite density was determined by land use only (i.e., no effect of vegetation productivity), the global total emission in 2020 was estimated as 15.6 ± 7.1 Tg CH_4_ year^−1^, that is, 6% higher than the control estimate of this study. In this case, the uniform termite densities (8 g dry weight m^−2^ in tropical deciduous forests and 11 g dry weight m^−2^ in tropical evergreen forests, after Sanderson, 1996^31^) applied to tropical ecosystems, resulted in a higher global total value. Third, combining the first and second cases, the global termite emission was estimated as 19.2 ± 3.0 Tg CH_4_ year^−1^, that is, 30% higher than the control estimate of this study. However, this third estimate is close to those of Fung et al.^14^ and Sanderson^31^, who used land use-specific termite density and emission factors. Thus, the selection of the emission factor dataset and of the termite-density mapping method can explain a large part of the disparity among the previous studies, excepting the extremely low or high ones certainly attributable to the use of biased data. Remarkably, the estimation procedures also affected the temporal trend of termite emissions. When only climate and land-use effects were included (i.e., the effect of vegetation productivity was ignored), global termite biomass and CH_4_ emissions were estimated to gradually decrease through time because of deforestation in tropical areas and resultant habitat loss (Fig. 2). In the future, the estimation uncertainty is expected to be reduced through the accumulation of additional field and laboratory measurement data, improved upscaling that takes account of the spatial representativeness of data and the determining mechanisms, and verification using independent evidence.

### Global CH_4_ budget and termite emissions

The estimated global total emission in 2020, 14.8 ± 6.7 Tg CH_4_ year^−1^, confirms that termite emissions constitute a substantial component of the global CH_4_ budget: about 2% of the global total (natural + anthropogenic) emissions and 4% of natural emissions^5,32^. The total termite emission is larger than anthropogenic emissions from most countries (except China, India, United States, Brazil, Russia, and Indonesia) and comparable to emissions from paddy fields in East Asia^33^.

Termite emission is one of the major emission sources in upland areas (other sources: wildfires, wild animals, and geological processes). Indeed, termite emissions can turn many uplands into net CH_4_ sources, even after uptake by soil methanotrophic oxidation is subtracted (Fig. 1c). However, the emissions were generally weak in their intensity (on the order of 0.1 g CH_4_ m^−2^ year^−1^; Fig. 1b) and distributed over a vast area of uplands; as a result, it is difficult to detect and quantify the signal using atmospheric observations made from, for example, tall towers and satellites. Also, because the emissions are produced by common microbe taxa, the stable carbon isotope ratio of termite-emitted CH_4_ (δ^13^C-CH_4_, − 63.4 ± 6.4‰) is not distinguishable from that of CH_4_ emitted from wetlands and enteric fermentation^34^. These considerations suggest that a bottom-up approach is needed, but they also indicate the importance of taking termite emissions into account when evaluating national and regional CH_4_ budgets (e.g., Ito et al.^33^).

The results of this study imply that, in the future, termite emissions will increase globally (by 0.5–5.9 Tg CH_4_ year^−1^ by the end of this century), as a result of rising atmospheric CO_2_ and climate change. The poleward expansion of potential termite habitat is projected to result in additional CH_4_ emissions from temperate to boreal regions, even under the mitigation-oriented ssp126 scenario (Fig. 3). Although the projected magnitude of the change in termite emissions is smaller than the projected magnitude of the change in wetland emissions (+ 20 to 150 Tg CH_4_ year^−1^)^28^, the increase of termite emissions may have significance for regional and global CH_4_ budgets and climatic change. Based on the 20- or 100-year horizon Global Warming Potential values (79.7 and 27.0, respectively; after IPCC, 2021^3^), the increase of termite emissions corresponds to CO_2_ emissions of 4–129 Tg C year^−1^. The increase in termite emissions can, thus, substantially influence efforts to mitigate climatic change through emission reduction, especially under the Global Methane Pledge, which calls for country-level CH_4_ emissions to be reduced by 30% by 2030.

Clearly, when considering ecosystem management and climatic mitigation, we should note that the impacts of land use and climatic changes are complicated and interconnected. Land-use conversion for food and bioenergy production, especially in tropical regions, should suppress the emission increase to some extent. This may not be a main factor driving land-use decisions, because land-use conversion has stronger impacts on the CO_2_ budget and, possibly, biodiversity. Although not explored in this study, extreme climate events associated with climatic warming may affect termite activities and perhaps ecosystem integrity. The results presented in this study have implications for ecosystem management that considers the overlooked effects of decomposers and the non-CO_2_ greenhouse gas budget.

### Limitations and future perspectives

This study revisited global termite CH_4_ emissions, and it provides the first estimation of the temporal changes, but it has several limitations. First, up-to-date datasets were used, the spatial distributions of termite density and emission factors were not spatially resolved with high reliability. A new dataset compiled by Zhou et al.^20^ was used to capture the frequency distribution of termite emission factors, but the dataset does not differentiate among regional and phylogenetic groups^17,35,36^. Similarly, the present study did not treat soil-feeding (humivorous) and wood-feeding termites separately, although the former is reported to release a larger amount of CH_4_^23^. Further accumulation of observational data and analyses is required to fully characterize the spatial patterns of termites’ functional attributes. Second, the time lag in termite migration was not considered; instead, was implicitly assumed that termites are sufficiently mobile (through dispersion by flight or marching)^11^ to keep up with the habitat expansion caused by climatic warming. Several genetic and conservation studies have reported historical biogeographic aspects of termites^37,38^, but no direct observations of temporal changes in termite density in primary and secondary ecosystems is available (Fig. 2b). It is still uncertain whether termites can adapt to future climate change, which is predicted to proceed at unprecedented rates; therefore, the results of the present study (Fig. 3) likely show only the potential response. Third, actual ecological interactions are likely to be much more complicated than those included in this study. Termites can affect ecosystem structure and functions by altering carbon and nutrient cycles, while at the same time being themselves influenced by changes in vegetation and natural enemies. For example, Ashton et al.^39^ reported that termite abundance increases during droughts and that termites in tropical forests show higher drought resistance because of accelerated decomposition and altered soil properties. In addition, da Cunha et al.^40^ showed host plant differences influence the geographic distribution of wood-feeding termites. Furthermore, several ant species are termite predators, and their abundance thus affects termite density^41^. These ecological interactions might affect the termite habitat, diversity, and functions under changing environments, and detailed studies are needed to elucidate these mechanisms. To date, no terrestrial carbon cycle model or dynamic vegetation model, especially among those embedded in Earth system models, explicitly includes termite-driven processes^42^. Considering the extensive distribution, biomass, and dynamic flows of termites, it would be meaningful for these models to include termite-related factors. Their inclusion would surely result in improved reliability and ability to capture biogeochemical feedbacks.

## Methods and data

In this study, global termite CH_4_ emissions were estimated using empirical approaches adopted in previous studies and updated data of climate, land-use and land-cover, and termite distribution and emission factors. Also, the use of a process-based biogeochemical model (VISIT: Vegetation Integrated SImulator for Trace gases^33^) that simulates vegetation productivity and soil carbon cycle, allowed including environmental responses in a mechanistic manner.

### Geographic distribution of termites

Termite habitat area was assumed to be limited by temperature, while rainfall may affect termite diversity within tropical habitats^43,44^. To find a termite threshold of potential termite habitat, two global datasets of field-observed termite colonies were examined.

**Figure 4:**
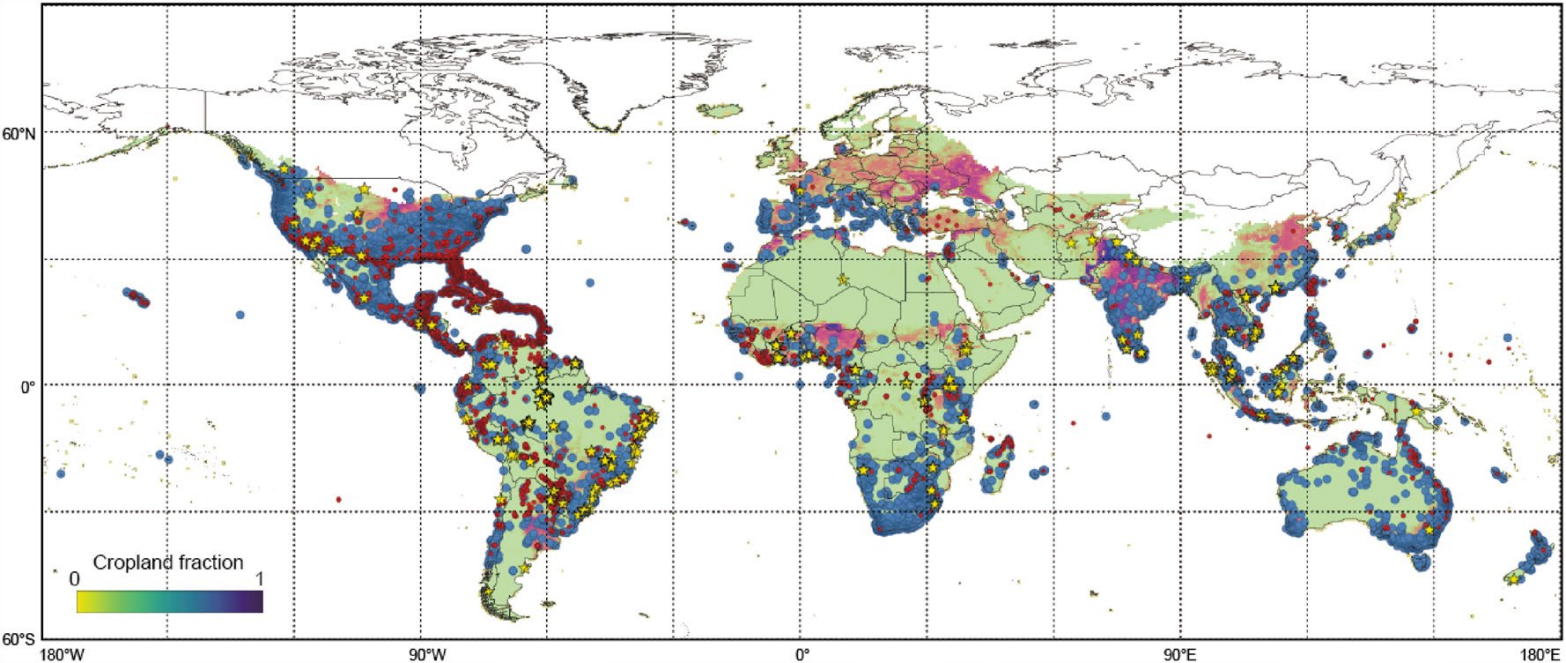
Distribution of observed termite colonies and potential habitat. Small red dots, University of Florida Termite Collection dataset; large blue dots, iNaturalist dataset; yellow stars, literature data. Areas with a minimum monthly mean temperature higher than − 8 °C (climatological mean) are colored by cropland fraction (Supplementary Fig. S1b) (Map generated by QGIS 3.28.7, https://qgis.org/en/site/).

- the University of Florida Termite Collection (small red dots in Fig. 4) (UFTC: https://www.termitediversity.org/), an open dataset containing more than 45,000 records of termite colonies, mainly from the Americas
- the iNaturalist dataset (large blue dots in Fig. 4) (https://www.inaturalist.org/), a community database of biodiversity containing > 24,000 records of termite observations.

For each dataset, records that included latitude and longitude values were used. Additionally, several studies in the literature were included (yellow stars in Fig. 4): Pullan^45^ for Africa, Palin et al.^46^ for the Amazon–Andes area, Jamali et al.^47^ for *Eucalyptus* forests in Australia, and Sheffrahn et al.^48^ for global highland observations.

Based on the observed termite distribution, a temperature threshold below which termites cannot survive over winter was examined. A global gridded dataset of historical climate conditions produced by the Climate Research Unit (CRU) TS4.05^49^ was used to derive mean monthly temperatures for 2001–2020. The climatic envelope of termite habitat, which encompasses most of the observed termite colonies and captures well their distribution boundaries, was examined. On the basis of many trials, a minimum monthly temperature higher than − 8 °C (Fig. 4) was selected as the temperature threshold explaining the observed termite distribution. Using this threshold, potential termite habitat was estimated annually during the study period.

### Termite CH_4_ emission

Termite CH_4_ emission at an arbitrary point (µg CH_4_ m^−2^ h^−1^) is calculated as follows:

1 $${\text{Emission }} = {\text{ Termite biomass density }} \times {\text{ Emission factor}}.$$

Biomass density of termites (g termite m^−2^) within the climatic envelope, as described in the previous section, was first estimated on the basis of land-cover type, as in previous studies^14^. This study referred to Sanderson^31^ for the mean termite density of each land-cover type; this value ranged from 0 g m^−2^ in tundra and polar desert to 11 g m^−2^ in tropical evergreen forest (Table 1). For cropland, the termite density and emission factor indicated in Table 1 were used in all cases. The global distribution of natural vegetation types was derived from Ramankutty and Foley^50^, and the historical change in cropland was derived from the Land Use Harmonization version 2 dataset (LUH2)^51^, because the expansion of cropland at the expense of natural vegetation can result in lower termite densities in tropical regions (Table 1). In tropical ecosystems, changes in termite density in response to vegetation productivity were included by using the relationship in Kirschke et al.^52^ and Saunois et al.^5^:

2 $${\text{Termite biomass density }} = { 1}.{21 }\cdot{\text{ exp}}\left( {0.000{\text{8 GPP}}} \right)$$

where GPP is annual gross primary production (g C m^−2^ year^−1^), estimated in this study using the VISIT model^33,53^. This empirical relationship indicates that termite biomass increases as the dry matter supply from vegetation increases.

**Table 1:** Land-cover/use type-specific emission factors and termite biomass density after Sanderson^31^.

Land-use/cover type	Emission factors (µg CH_4_ g^−1^ termite h^−1^)	Termite density (g m^−2^)
Tropical evergreen forest/woodland	5.9	11
Tropical deciduous forest/woodland	5.9	8
Temperate broadleaf evergreen forest/woodland	1.77	3
Temperate needleleaf evergreen forest/woodland	1.77	3
Temperate deciduous forest/woodland	1.77	3
Boreal evergreen forest/woodland	1.77	3
Boreal deciduous forest/woodland	1.77	3
Evergreen/deciduous mixed forest/woodland	1.77	3
Savanna	5.175	5.78
Grassland/steppe	1.77	5.2
Dense shrubland	5.1	8.43
Open shrubland	5.25	0.98
Tundra	0	0
Desert	1	3.1
Polar desert/rock/ice	0	0
Cropland	3.45	3.815

**Figure 5:**
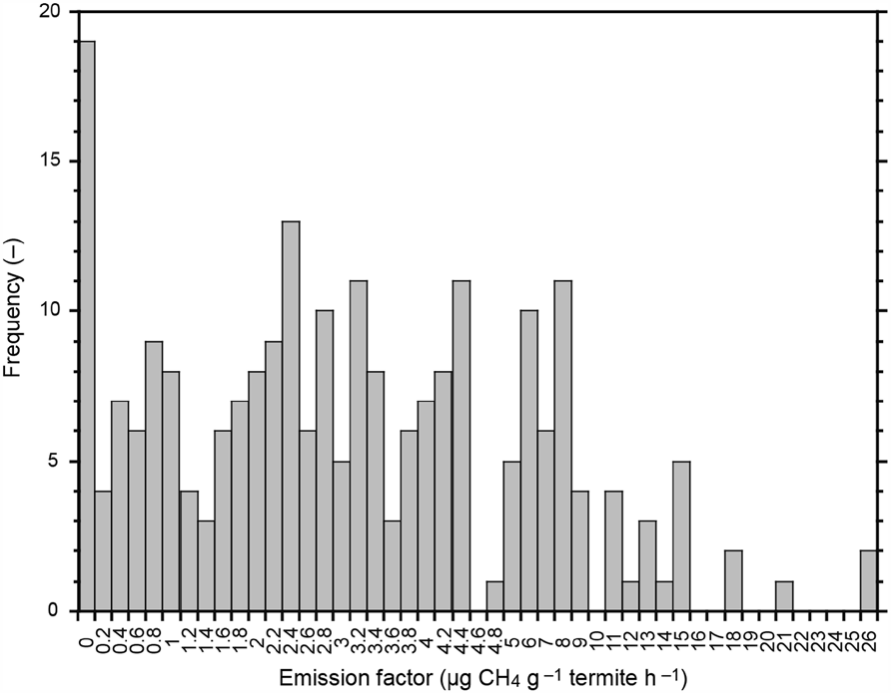
Frequency distribution of termite CH_4_ emission factors. Based on data from Zhou et al.^20^.

Emission factors (µg CH_4_ g^−1^ termite h^−1^) for each land-cover type were determined by sampling from the observed frequency distribution (Fig. 5) in a recent dataset compiled by Zhou et al.^20^; these values ranged from 0.0 to 25.26 µg CH_4_ g^−1^ termite h^−1^ (mean ± s.d., 3.81 ± 4.10 µg CH_4_ g^−1^ termite h^−1^). Annual emission was obtained simply by multiplying 24 (hours per day) and 365 (days per year). To assess the estimation uncertainty, alternative emission factor values (Table 1) were obtained from Sanderson^31^ and used for comparison estimation.

### Global estimations

Global estimations were conducted at a spatial resolution of 0.5° latitude × 0.5° longitude. Termite CH_4_ emission in each grid within the temperature threshold was estimated on the basis of land use, climate, vegetation productivity, and emission factors. At each grid, total emission was obtained as a sum of emissions from natural ecosystem and cropland weighted by cropland areal fraction. For the historical period from 1901 to 2021, climate (CRU TS4.06) and land use (LUH2) data were used to run the VISIT model and then to estimate termite CH_4_ emissions. Each simulation began with a spin-up iteration under stationary climate and land-use conditions until an equilibrium state of the carbon budget (annual change in ecosystem carbon stock of < 0.001 Mg C ha^−1^ year^−1^) was reached in each grid: 300–4000 years depending on the conditions. The annual GPP estimated by the VISIT model was then used to estimate termite density in tropical ecosystems; for other ecosystems, fixed land use-specific termite densities were used. For upland aerobic soils, oxidation of CH_4_ by methanotrophic microbes was also estimated by the VISIT model^53^, based on soil diffusivity and temperature.

For the future period until 2100, scenario-based land use and model-projected climate data were used. Two Shared Socioeconomic Pathways (land-use and atmospheric CO_2_ and CH_4_ concentration scenarios), ssp126 and ssp585, were used^54^. These pathways assumed a mitigation-oriented and adaptation-oriented society, respectively, along with corresponding climate projections by five climate models (GFDL-ESM4, IPSL-CM6A-LR, MPI-ESM1-2-HR, MRI-ESM2-0, UKESM1-0-LL) from the Coupled Model Intercomparison Project phase 6 (CMIP6)^55^. The VISIT-estimated GPP was similarly used to estimate termite density in tropical ecosystems.

### Statistical analysis of uncertainty

Because the observed emission factors range widely (Fig. 5), the range of estimation uncertainty was estimated by conducting 1,000 ensemble calculations of the emissions using emission factors randomly sampled from the observed frequency distribution (Fig. 5). Furthermore, the uncertainty associated with the estimation method was examined by comparing with the estimate using emission factors prescribed for each land-cover type (Table 1).

### Supplementary Information

Supplementary Information.

## Data Availability

Data relevant to this study are available from the data repository of the National Institute for Environmental Studies (NIES): https://www.nies.go.jp/doi/10.17595/20210521.001-e.html. Climate data is publicly available: https://crudata.uea.ac.uk/cru/data/hrg/. Laud use data is publicly available: https://luh.umd.edu/. CMIP6 data produced by GFDL-ESM4, IPSL-CM6A-LR, MPI-ESM1-2-HR, MRI-ESM2-0, and UKESM1-0-LL are licensed under a Creative Commons Attribution 4.0 International License (CC BY 4.0; https://creativecommons.org/licenses/) and available from https://pcmdi.llnl.gov/CMIP6/.

## Code availability

The code used for estimating the emission and sample output are available from: 10.5281/zenodo.8145701.
