# Supplementary material for: Global termite methane emissions have been affected by climate and land-use changes
Source: Sci Rep. 2023 Oct 11;13:17195. doi: 10.1038/s41598-023-44529-1 (PMC10567709; doi:10.1038/s41598-023-44529-1)
Supplement: Supplementary file 1 — Supplementary Information. [file 41598_2023_44529_MOESM1_ESM.docx]

**Supplementary information**

**Global termite methane emissions have been affected by climate and land-use changes**

Akihiko Ito ^1,2,3^

^1^ University of Tokyo, Tokyo

^2^ National Institute for Environmental Studies, Tsukuba

^3^ Japan Agency for Marine-Earth Science and Technology, Yokohama

Correspondence

Akihiko Ito (e-mail: akihikoito@g.ecc.u-tokyo.ac.jp)

| **Supplementary Table S1.** Summary of global termite CH_4_ emissions reported by previous studies. | | |
| --- | --- | --- |
| Reference | Tg CH_4_ yr^–1^ | Notes |
| Zimmerman et al. (1982) ^1^ | 151.6 | Based on carbon consumption |
| Rasmussen and Khalil (1983) ^2^ | 50 (10–100) | Based on termite population |
| Seiler et al. (1984) ^3^ | 2–5 | Mound emissions, based on CH_4_/CO_2_ ratios |
| Fraser et al. (1986) ^4^ | 14 (6–42) | Based on global termite biomass |
| Khalil et al. (1990) ^5^ | 12 (2–20) | Mound emissions, based on CH_4_/CO_2_ ratios |
| Fung et al. (1991) ^6^ | 20 | Based on land use |
| Martius et al. (1993) ^7^ | 26 | Based on global termite biomass, mound emissions |
| Sanderson (1996) ^8^ | 19.7 ± 1.5 | Based on global termite biomass |
| Bignell et al. (1997) ^9^ | 17–96 | Based on global termite biomass |
| Sugimoto et al. (1998) ^10^ | 1.5–7.4 | Based on global termite biomass |
| Brümmer et al. (2009) ^11^ | 0.9 | Based on mound density |
| Kirschke et al. (2013) ^12^ | 8.7 ± 3.1 | Based on global termite biomass |
| Saunois et al. (2016), Saunois et al. (2020) ^13,14^ | 9 (3–15) | Based on global termite biomass |

**
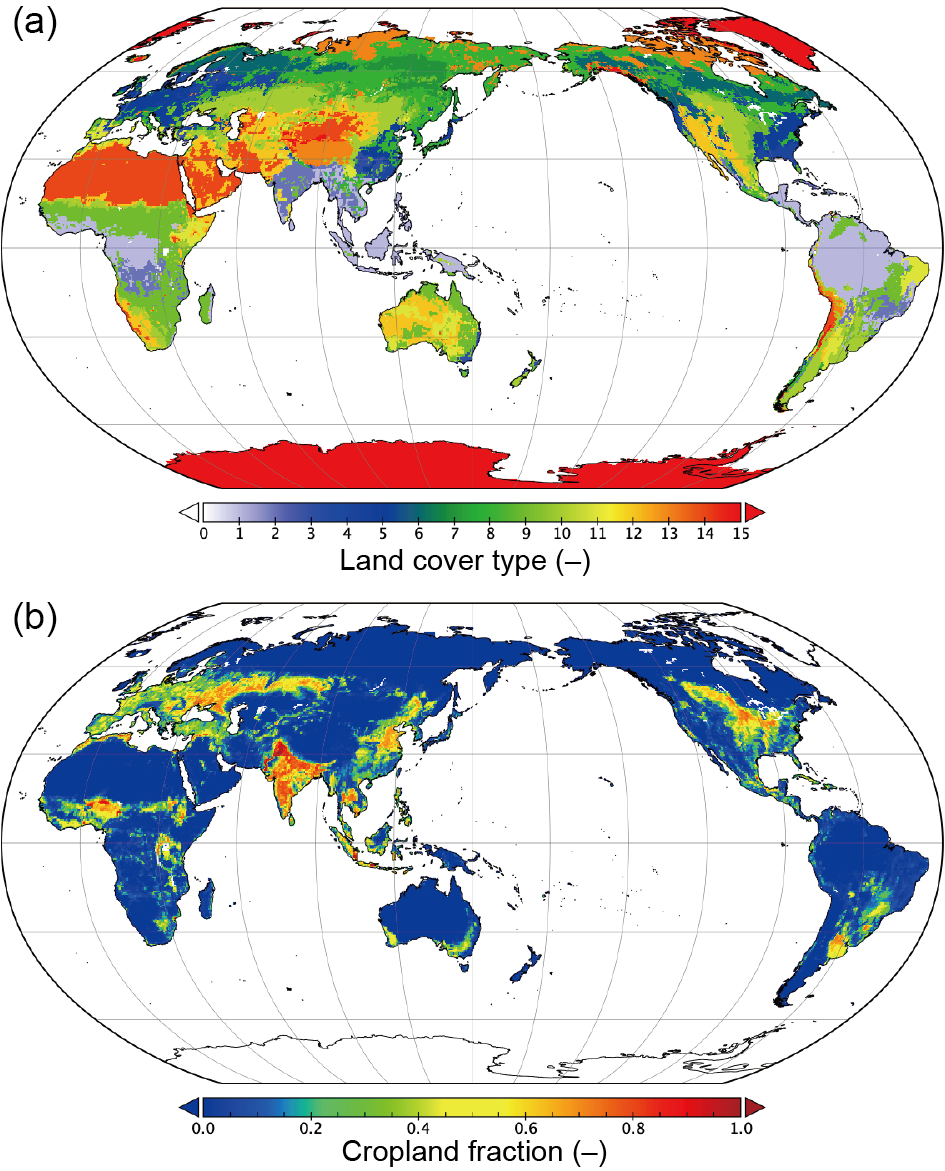
**

**Supplementary Fig. S1 | Land-cover maps used in this study.** (a) Potential vegetation and (b) cropland fraction in 2020. Vegetation types in (a): 1, tropical evergreen forest/woodland; 2, tropical deciduous forest/woodland; 3, temperate broadleaf evergreen forest/woodland; 4, temperate needleleaf evergreen forest/woodland; 5, temperate deciduous forest/woodland; 6, boreal evergreen forest/woodland; 7, boreal deciduous forest/woodland; 8, evergreen/deciduous mixed forest/woodland; 9, savanna; 10, grassland/steppe; 11, dense shrubland; 12, open shrubland; 13, tundra; 14, desert; 15, polar desert/rock/ice. (Maps generated by Panoply 5.2.9, https://www.giss.nasa.gov/tools/panoply/).

**
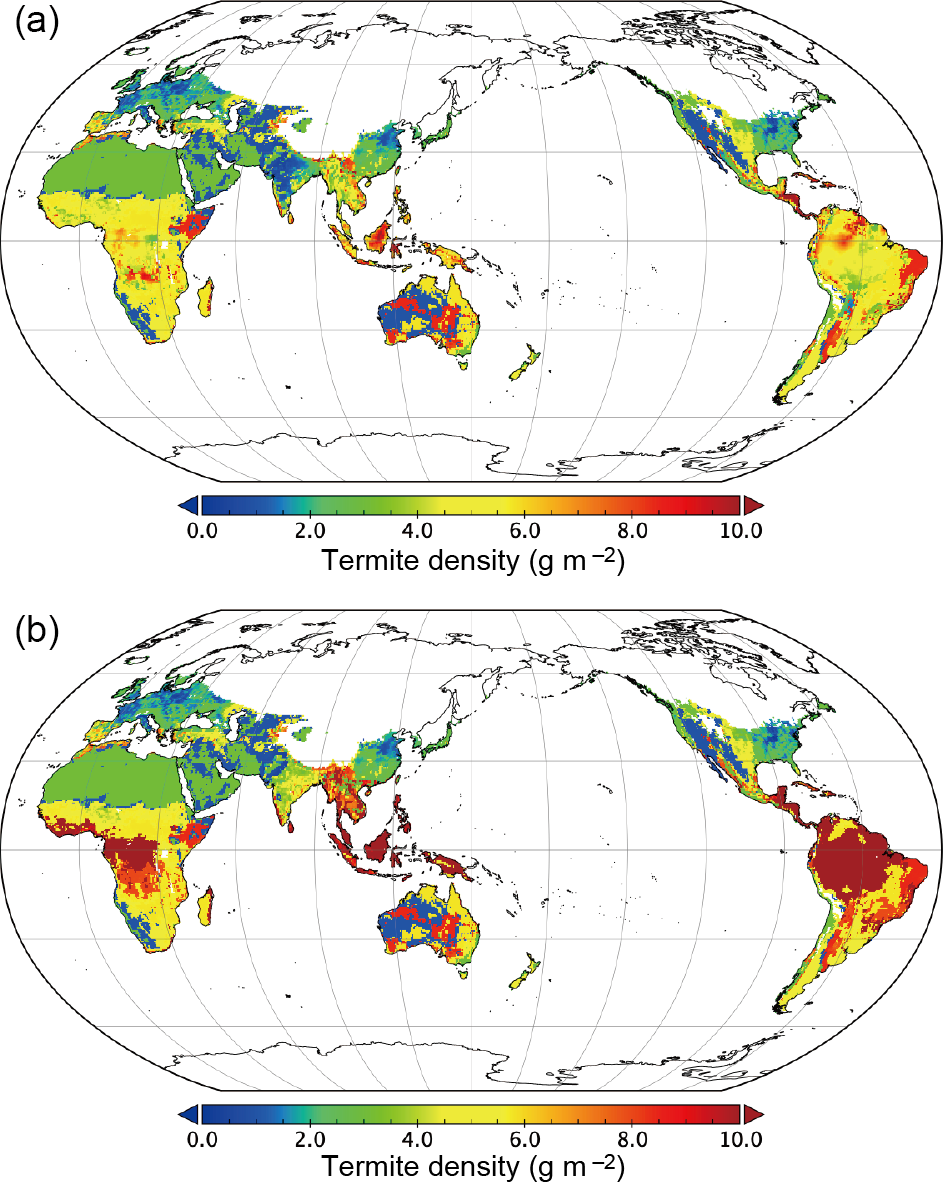
**

**Supplementary Fig. S2 | Termite density estimated in this study.** (a) Based on land use and vegetation productivity in the tropics, and (b) based on land cover only. (Maps generated by Panoply 5.2.9, https://www.giss.nasa.gov/tools/panoply/).


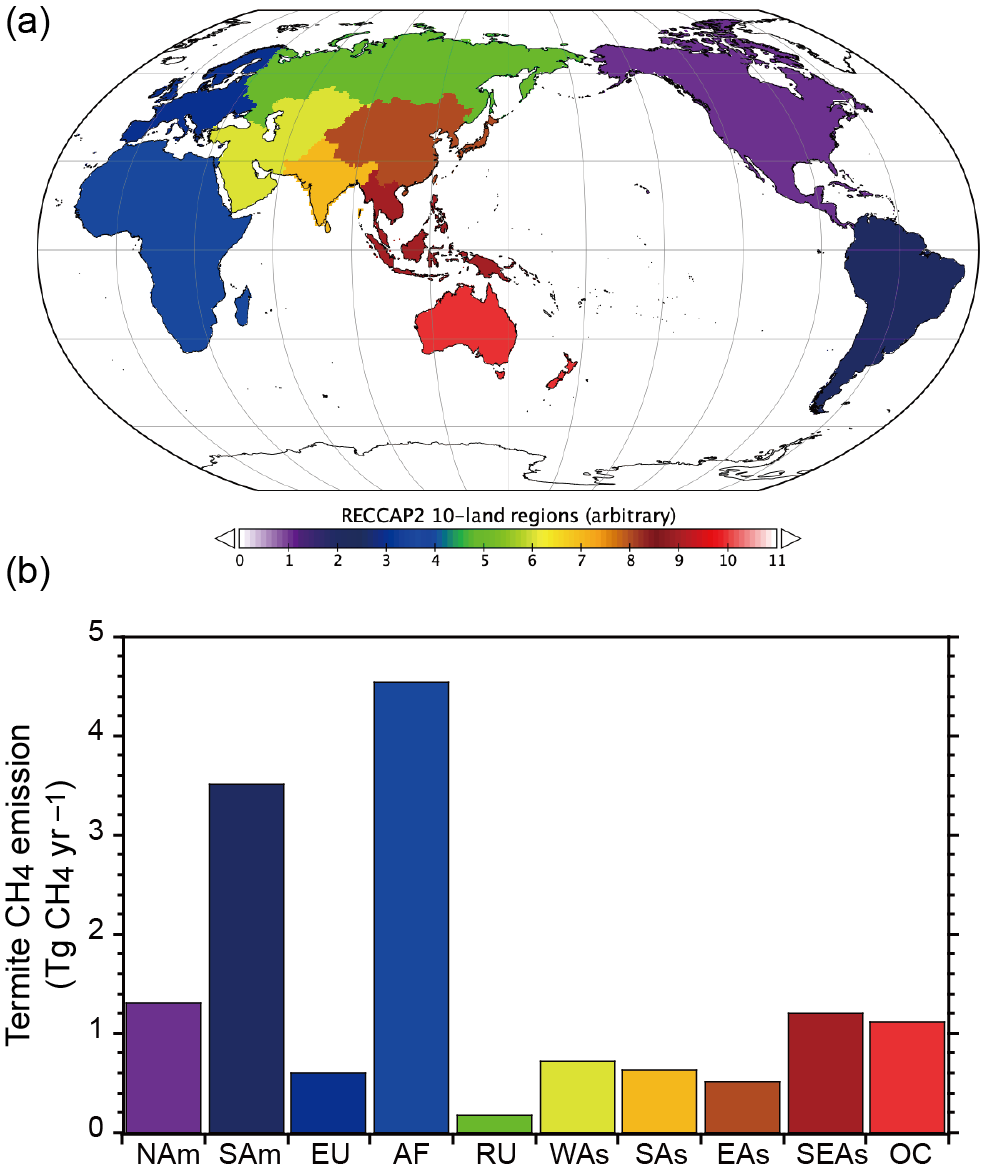


**Supplementary Fig. S3 | Regional termite CH_4_ emissions in 2020.** (a) Map of the land regions. (b) Estimated termite CH_4_ emissions in 2020 in each land region. NAm, North America; SAm, South America; EU, Europe; AF, Africa; RU, Russia; WAs, West Asia; SAs, South Asia; EAs, East Asia; SEAs, Southeast Asia; OC, Oceania. (Maps generated by Panoply 5.2.9, https://www.giss.nasa.gov/tools/panoply/).


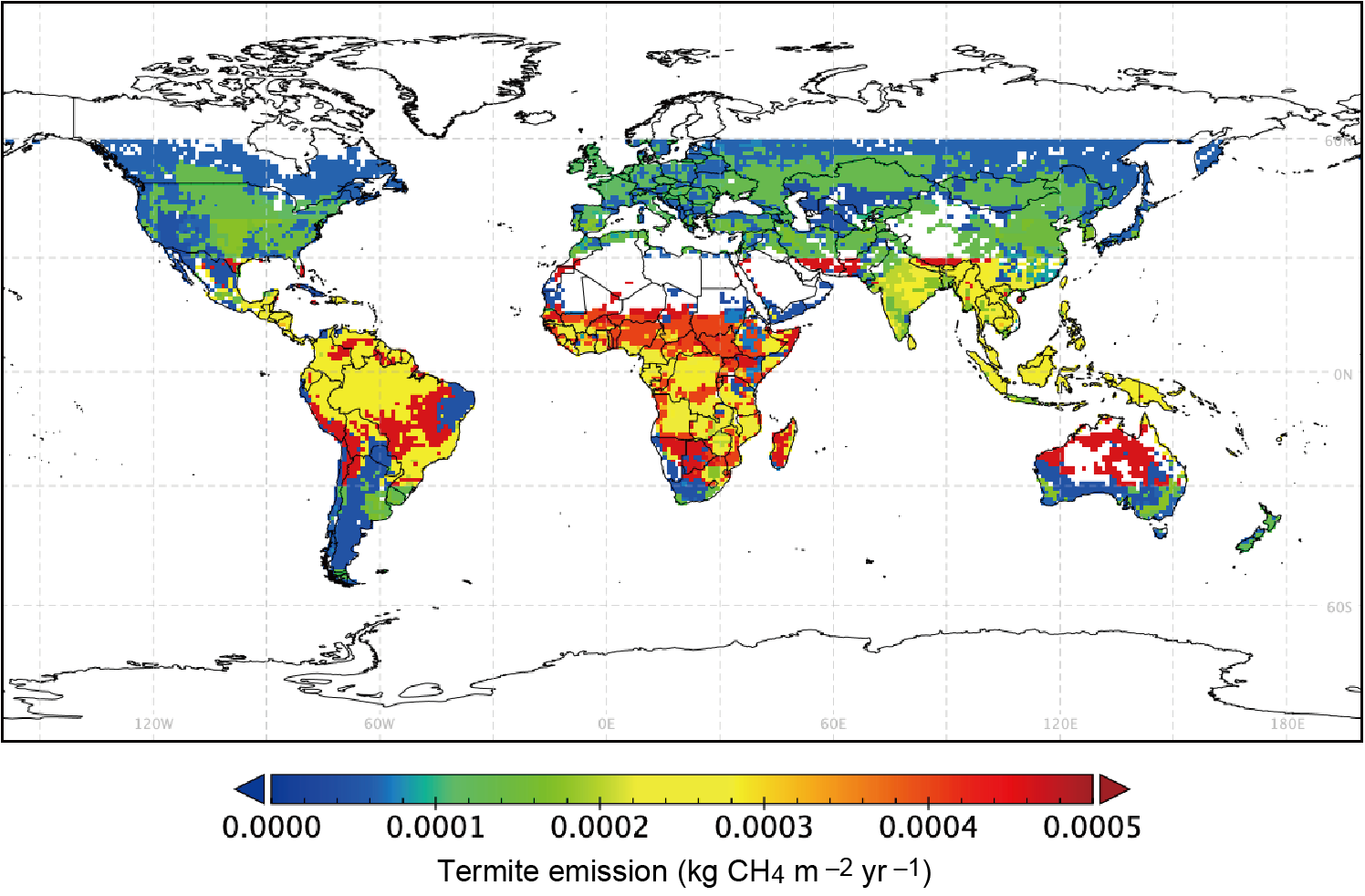


**Supplementary Fig. S4 | Global distribution of termite CH_4_ emissions as estimated by Fung et al.** **(1991) ^6^.** Numerical data obtained from https://pubs.giss.nasa.gov/abs/fu08000d.html). (Maps generated by Panoply 5.2.9, https://www.giss.nasa.gov/tools/panoply/).


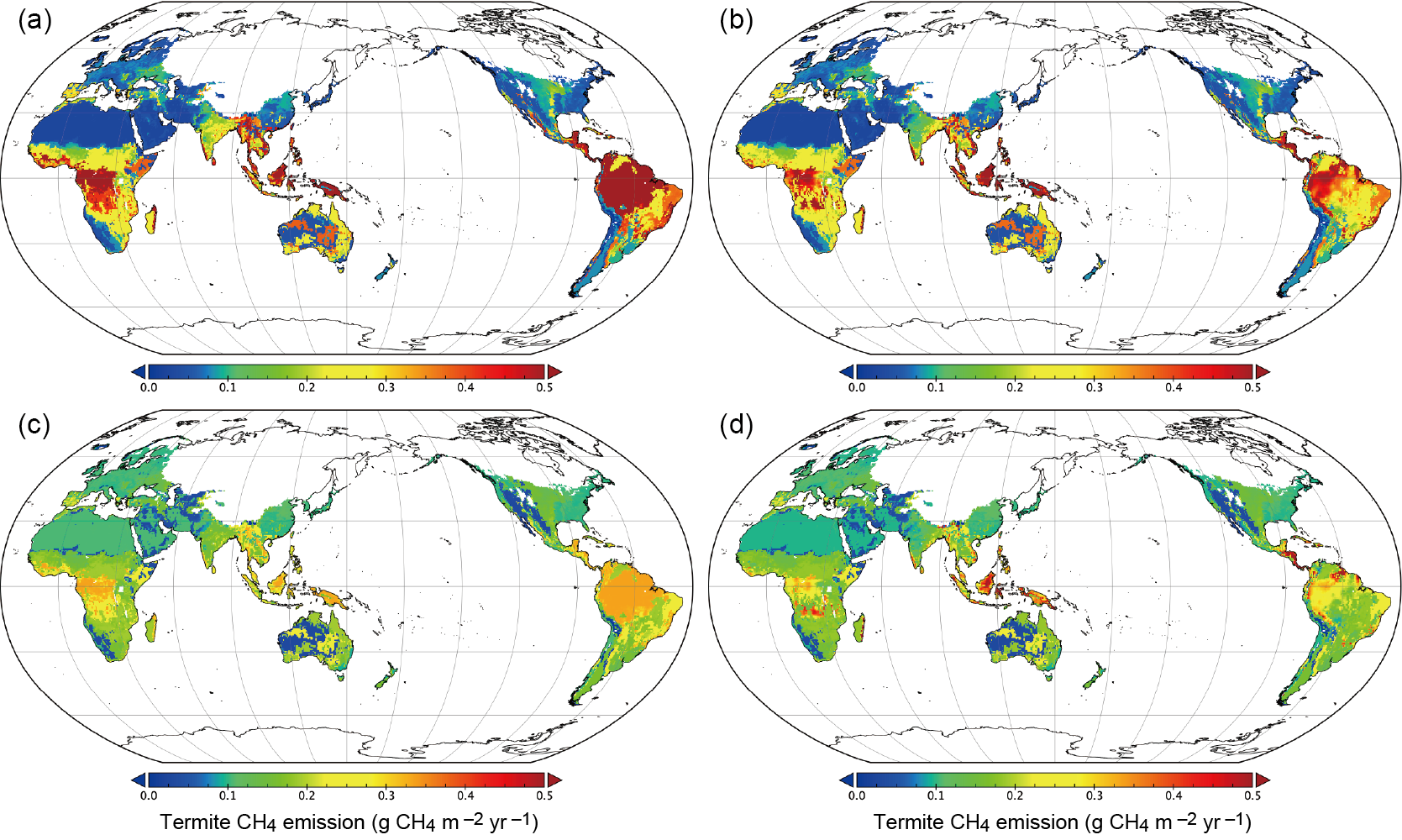


**Supplementary Fig. S5 | Estimated termite CH_4_ emissions in 2020.** Estimates made by using (a) land use-based termite density and emission factors from Sanderson (1996) ^8^, (b) land use- and vegetation productivity-based termite density and emission factors from Sanderson (1996) ^8^, (c) land use-based termite density and emission factors from Zhou et al. (2023) ^15^, and (d) land use- and vegetation productivity-based termite density and emission factors from Zhou et al. (2023) ^15^ (same as Fig. 1b and referred as the control estimate in text). (Maps generated by Panoply 5.2.9, https://www.giss.nasa.gov/tools/panoply/).

**References**

1 Zimmerman, P. R., Greenberg, J. P., Wandiga, S. O. & Crutzen, P. J. Termites: A potentially large source of atmospheric methane, carbon dioxide, and molecular hydrogen. *Science* **218**, 563-565 (1982).

2 Rasmussen, R. A. & Khalil, M. A. K. Global production of methane by termites. *Nature* **301**, 700-702 (1983).

3 Seiler, W., Conrad, R. & Scharffe, D. Field studies of methane emission from termite nests into the atmosphere and measurements of methane uptake by tropical soils. *Journal of Atmospheric Chemistry* **1**, 171–186 (1984).

4 Fraser, P. J., Rasmussen, R. A., Creffield, J. W., French, J. R. & Khalil, M. A. K. Termites and global methane - another assessment. *Journal of Atmospheric Chemistry* **4**, 295-310 (1986).

5 Khalil, M. A. K., Rasmussen, R. A., French, J. R. J. & Holt, J. A. The influence of termites on atmospheric trace gases: CH_4_, CO_2_, CHCl_3_, N_2_O, CO, H_2_, and light hydrocarbons. *Journal of Geophysical Research* **95**, 3619–3634 (1990).

6 Fung, I. *et al.* Three-dimensional model synthesis of the global methane cycle. *Journal of Geophysical Research* **96**, 13033-13065 (1991).

7 Martius, C. *et al.* Methane emission from wood-feeding termites in Amazonia. *Chemosphere* **26**, 623-632 (1993).

8 Sanderson, M. G. Biomass of termites and their emissions of methane and carbon dioxide: A global database. *Global Biogeochem. Cycles* **10**, 543–557 (1996).

9 Bignell, D. E., Eggleton, P., Nunes, L. & Thomas, K. L. in *Firests and Insects* (eds A.D. Watt, N.E. Stork, & M.D. Hunter) 109–134 (Chapman and Hall, 1997).

10 Sugimoto, A., Inoue, T., Kirtibutr, N. & Abe, T. Methane oxidation by termite mounds estimated by thye carbon isotopic composition of methane. *Global Biogeochem. Cycles* **12**, 595-605 (1998).

11 Brümmer, C., Papen, H., Wassmann, R. & Brüggemann, N. Fluxes of CH4 and CO_2_ from soil and termite mounds in south Sudanian savanna of Burkina Faso (West Africa). *Global Biogeochem. Cycles* **23**, 10.1029/2008GB003237 (2009). https://doi.org/10.1029/2008GB003237

12 Kirschke, S. *et al.* Three decades of global methane sources and sinks. *Nature Geoscience* **6**, 813–823 (2013). https://doi.org/10.1038/NGEO1955

13 Saunois, M. *et al.* The global methane budget: 2000–2012. *Earth System Science Data* **8**, 697–751 (2016). https://doi.org/10.5194/essd-8-697-2016

14 Saunois, M. *et al.* The global methane budget 2000–2017. *Earth System Science Data* **12**, 1561–1623 (2020). https://doi.org/10.5194/essd-12-1561-2020

15 Zhou, Y., Staver, A. C. & Davies, A. B. Species-level termite methane production rates. *Ecology* **104**, e3905 (2023). https://doi.org/10.1002/ecy.3905
